# Validation of birth certificate and maternal recall of events in labor and delivery with medical records in the Iowa health in pregnancy study

**DOI:** 10.1186/s12884-022-04581-7

**Published:** 2022-03-22

**Authors:** Christina Ziogas, Jenna Hillyer, Audrey F. Saftlas, Cassandra N. Spracklen

**Affiliations:** 1 Department of Biostatistics and Epidemiology, University of Massachusetts Amherst, 715 North Pleasant Street, 429 Arnold House, MA 01003 Amherst, USA; 2 Department of Epidemiology, University of Iowa, Iowa City, IA 52399 USA; 3 Child Advocacy Studies Program, College of Education, Winona State University, Winona, MN 55987 USA

**Keywords:** Validation, Validation study, Birth certificate, Medical records, Pregnancy, Child birth

## Abstract

**Background:**

Epidemiological research of events related to labor and delivery frequently uses maternal interview or birth certificates as a primary method of data collection; however, the validity of these data are rarely confirmed. This study aimed to examine the validity of birth certificate data and maternal interview of maternal demographics and events related to labor and delivery with data abstracted from medical records in a US setting.

**Methods:**

Birth certificate and maternal recall data from the Iowa Health in Pregnancy Study (IHIPS), a population-based case-control study of risk factors for preterm and small-for-gestational age births, were linked to medical record data to assess the validity of events that occurred during labor and delivery along with reported maternal demographics. Sensitivity, specificity, positive and negative predictive values, and kappa scores were calculated.

**Results:**

Postpartum maternal recall and birth certificate data were excellent for infant characteristics (birth weight, gestational age, infant sex) and variables related to labor and delivery (mode of delivery) when compared with medical records. Birth certificate data for labor induction had low sensitivity (46.3%) and positive predictive value (18.3%) compared to medical records. Compared to maternal interview, birth certificate data also had poor agreement for smoking and alcohol use during pregnancy. Agreement between all three methods of data collection was very low for pregnancy weight gain (kappa = 0.07-0.08).

**Conclusions:**

Maternal interview and birth certificate data can be a valid source for collecting data on infant characteristics and events that occurred during labor and delivery. However, caution should be used if solely using birth certificate data to gather data on maternal demographic and/or lifestyle factors.

## Background

Epidemiological research of events related to labor and delivery frequently uses maternal self-report from interviews and/or questionnaires as the primary method of data collection. Although medical records are often considered to be the most accurate source of information, collecting self-report data is typically faster and less expensive than other methods of data acquisition. Further, medical records often do not contain information outside of medical diagnoses, procedures, and test results that were obtained during the prenatal visits and/or labor and delivery, including maternal stress and experiences, lifestyle habits, and/or domestic abuse. Other existing data sources, such as birth certificates, can also be used as a means for data collection and are also used frequently to identify potential study participants. However, despite the widespread use of both birth certificate data and data from maternal interviews, the validity of the data collected is rarely examined within a study population.

To ensure data accuracy across all three sources, further research is necessary in order to observe how one set of data compares to the others. Previous studies have shown that the validity of maternal recall data against data from the medical records varies by the type of information, ranging from very good for infant characteristics such as birth weight [1–6] and gestational age [4–8] to satisfactory or poor for events occurring in labor and delivery [6, 9]. Importantly, recall time since birth has been shown to affect the validity estimates [3, 6, 10]. However, few validation studies have been performed in populations within the United States [1, 3, 4, 10, 11] and little research has been published examining the validity of birth certificate data to either maternal interview [11–13] or medical records [14, 15].

The purpose of this study was to examine the validity of birth certificate data and data from maternal interviews for events related to labor and delivery with data abstracted from medical records using data from the Iowa Health in Pregnancy Study (IHIPS). This will help us gain further insight on the level of agreement between labor and delivery data from maternal interviews and birth certificates compared to their corresponding medical records.

## Methods

### Study population

The Iowa Health in Pregnancy Study (IHIPS) is a population-based case-control study designed to identify risk and protective factors associated with preterm (PTB) and small-for-gestational age (SGA) births [16, 17]. Participants were eligible for inclusion in IHIPS if they: 1) resided in one of the four Iowa counties included in the study, and 2) had a live birth between May 2002-June 2005. Briefly, an introductory letter was mailed to all potential case (PTB and SGA) and control participants identified from the Iowa electronic birth certificate file (*n* = 7202) followed by a phone call inviting them to be screened for eligibility (*n* = 4250 reached by telephone). Participants were excluded from the study if they met any of the following criteria: < 18 years of age at the time of delivery; non-English speaker; index pregnancy included twins or higher order birth; or if the woman had a prepregnancy diagnosis of type 1 or type 2 diabetes, systemic lupus, or chronic renal disease (*n* = 548 excluded). Those who were eligible provided verbal consent were asked to complete a 45-min computer-assisted telephone interview (CATI; *n* = 2709). Medical record review of the their prenatal and hospital delivery records to validate the preterm and SGA outcomes was completed for 72.9% of participants in this analysis. In total, data from at least two sources (birth certificates and maternal interview) and from all three sources (birth certificates, maternal interview, and medical records) were available for 2709 and 1976 women, respectively. Of note, the IHIPS study was conducted before it was common practice to ask participants about their gender identification and pronoun preference. We refer to our participants as “women” throughout the manuscript, although we want to acknowledge that some of our participants may not identify themselves as such.

### Outcome definitions

We defined low birth weight (LBW) as any infant born weighing < 2500 g (< 5 lbs. 8 oz). Infants were considered preterm if they were born at < 37 weeks gestation and post-term if born at ≥41 weeks gestation. Gestational weight gain was defined as an increase in the woman’s weight from prepregnancy to the end of pregnancy (yes, gained weight/no, did not gain weight). Quantitative measures of gestational weight gain were not used due to the sparsity of available data.

### Birth certificate data

Birth certificate data were initially used to identify potential case and control study participants for IHIPS. Data from the birth certificates consisted of general information about the infant, demographic information about the mother and father (e.g., race/ethnicity, marital status), and information about the birth (e.g., day, time, location). Birth weight was reported in either grams or pounds and ounces, and gestational age was reported in weeks. Other data pertaining to the events of labor and delivery reported on the birth certificate include mode of delivery, Apgar score, and whether or not labor was induced.

### Maternal interview

Women who consented to participate in IHIPS completed a 45-min CATI survey that included questions related to her demographics, health history, reproductive history, pregnancy experiences with the index pregnancy, and her partner(s). Women were also asked to recall specific details about the index pregnancy, including the baby’s birth weight (pounds and ounces), sex (male/female), the gestational age (weeks), and events related to labor and delivery.

### Medical record abstraction

Among participants who consented to having their medical records reviewed, trained medical record abstractors reviewed prenatal and hospital delivery records. In addition to events from labor and delivery, available data pertaining to the woman’s reproductive history and prenatal care were also abstracted when available. Birth weight data was recorded in grams when possible. All gestational age values were abstracted in weeks; when the details were provided, we recorded the number of weeks + days.

### Statistical analyses

Validation statistics were calculated for the following comparisons: 1) birth certificate and maternal interview; 2) birth certificate and medical record; and 3) maternal interview and medical record. Validation of categorical variables was measured by calculating sensitivity, specificity, positive predictive value (PPV), negative predictive value (NPV), and kappa scores. The validity of continuous variables was determined by calculating the proportion of gestational age and birth weight measures that fell within one week and 50 g increments. Analyses were performed using SAS Studio version 3.8.

## Results

The prevalence (%) of each labor and delivery event in the maternal interview, birth certificate, and medical record data are shown in Table 1. Overall, the prevalence for most of the characteristics and events is similar between the data sources. However, the prevalence of labor induction is much lower among the birth certificates (21.8%) compared to the medical records (33.2%). Additionally, the prevalence of smoking and alcohol use during pregnancy differed between maternal interview and birth certificate data.

**Table 1:** Prevalence of labor and delivery characteristics from maternal interview, birth certificates, and medical record data, IHIPS

Characteristic	Maternal interview (***n*** = 2709)	Birth certificate (***n*** = 2709)	Medical records (***n*** = 1976)
Infant male sex	50.5	–	50.5
Low birth weight	19.8	17.8	21.7
Preterm birth	29.2	28.1	29.2
Post-term birth	9.8	9.7	10.4
Pregnancy weight gain	97.0	98.5	78.6
Vaginal delivery	73.8	72.9	72.7
Labor induction	–	21.8	33.2
Smoking during pregnancy	11.3	12.9	–
Alcohol use during pregnancy	5.5	1.0	–
Race/ethnicity			
Non-Hispanic white	88.4	90.2	–
Black	5.5	5.6	–
Asian	3.4	3.7	–
Hispanic	2.3	2.8	–

Each cell presents the percent prevalence of each characteristic within the data set. “- “indicates data were not available from a particular source

Tables 2, 3 and 4 show the overall agreement between birth certificate, maternal interview, and medical record data from IHIPS. Compared to medical records, maternal recall at an average of 9.6 months postpartum was found to be valid with high sensitivity, specificity, NPV, PPV, and kappa scores, for low birth weight, preterm birth, post-term birth, mode of delivery, and infant sex (Table 3). Birth certificates were also found to be valid when compared to the medical records for low birth weight, preterm birth, post-term birth, and mode of delivery (Table 4). However, birth certificates were only moderately accurate in reporting labor induction compared to medical records (kappa = 0.61; Table 4). Further, there was a lack of agreement between birth certificates and maternal interview in the reporting of smoking and alcohol use during pregnancy (kappa = 0.17 and 0.12, respectively).

**Table 2:** Validity of birth certificate data compared to maternal interview in the Iowa health in pregnancy study

Variable	Sensitivity (%)	Specificity (%)	PPV (%)	NPV (%)	True Positives (N)	False Positives (N)	False Negatives (N)	True Negatives (N)	Kappa
Low birthweight	95.8%	98.7%	94.0%	99.1%	437	28	19	2187	0.94
Preterm birth	99.6%	99.7%	99.2%	99.8%	755	6	3	1932	0.99
Post-term birth	98.9%	100.0%	100.0%	99.8%	260	0	3	1669	0.99
Pregnancy weight gain	99.2%	20.2%	97.6%	43.2%	2604	63	21	16	0.26
Vaginal delivery	99.1%	75.2%	91.6%	96.7%	1492	137	14	415	0.80
Smoking	10.6%	99.8%	90.2%	88.3%	37	4	313	2357	0.17
Alcohol use	34.6%	96.2%	8.2%	99.3%	9	101	17	2537	0.12
Race/ethnicity									
White	99.2%	91.7%	99.1%	92.4%	2347	22	20	244	0.91
Black	90.8%	99.6%	93.2%	99.4%	138	10	14	2457	0.91
Asian	91.9%	91.7%	97.9%	99.7%	91	2	8	2608	0.95
Hispanic	67.6%	99.6%	82.0%	99.1%	50	24	24	2618	0.74

Low birthweight was defined as infant weighing < 2500 g at birth. Preterm and post-term birth were defined as gestational age < 37 and ≥ 41 weeks gestation, respectively. Pregnancy weight gain was defined as an increase in maternal weight from the start of pregnancy through delivery; dichotomized as “yes” or “no”. Mode of delivery options were “vaginal” or “caesarean”

**Table 3:** Validity of maternal interview data compared to medical records in the Iowa health in pregnancy study

Variable	Sensitivity (%)	Specificity (%)	PPV (%)	NPV (%)	True Positives (N)	False Positives (N)	False Negatives (N)	True Negatives (N)	Kappa
Low birthweight	99.3%	98.4%	92.2%	99.5%	283	24	8	1488	0.94
Preterm birth	99.1%	99.6%	99.1%	99.6%	539	5	5	1389	0.99
Post-term birth	97.5%	100.0%	100.0%	99.6%	196	0	5	1194	0.99
Pregnancy weight gain	98.3%	100.0%	100.0%	3.7%	1509	0	26	1	0.07
Vaginal delivery	97.3%	97.9%	99.2%	93.0%	1383	11	39	516	0.94
Male sex	99.2%	99.0%	99.1%	99.1%	980	9	8	927	0.98

Low birthweight was defined as infant weighing < 2500 g at birth. Preterm and post-term birth were defined as gestational age < 37 and ≥ 41 weeks gestation, respectively. Pregnancy weight gain was defined as an increase in maternal weight from the start of pregnancy through delivery; dichotomized as “yes” or “no”. Mode of delivery options were “vaginal” or “caesarean”

**Table 4:** Validity of birth certificate data compared to medical records in the Iowa health in pregnancy study

Variable	Sensitivity (%)	Specificity (%)	PPV (%)	NPV (%)	True Positives (N)	False Positives (N)	False Negatives (N)	True Negatives (N)	Kappa
Low birthweight	98.3%	98.7%	93.5%	99.7%	289	20	5	1515	0.95
Preterm birth	99.3%	99.9%	99.8%	99.7%	543	1	4	1395	0.99
Post-term birth	97.5%	100.0%	100.0%	99.6%	196	0	5	1194	0.99
Pregnancy weight gain	98.9%	6.2%	79.5%	49.1%	1536	397	17	26	0.08
Vaginal delivery	97.3%	97.9%	99.2%	93.0%	1383	11	39	516	0.94
Induced labor	46.3%	80.0%	18.3%	93.9%	386	41	266	1213	0.61

Low birthweight was defined as infant weighing < 2500 g at birth. Preterm and post-term birth were defined as gestational age < 37 and ≥ 41 weeks gestation, respectively. Pregnancy weight gain was defined as an increase in maternal weight from the start of pregnancy through delivery; dichotomized as “yes” or “no”. Mode of delivery options were “vaginal” or “caesarean”

Tables 5 and 6 show the overall agreement between birth certificate, maternal interview, and medical record data from IHIPS when available data were examined as continuous variables. All three data sources were in very high agreement with respect to gestational age with nearly 99% exact agreement. For birthweight, there is high accuracy of maternal reporting (90.9%) and very high accuracy of the birth certificates (98.6%) within a small margin of error (± 50 g). When comparing birth certificate data to medical records, the birth certificate was highly accurate for 1-min and 5-min Apgar scores with 97.1 and 98.4% exact agreement, respectively.

**Table 5:** Continuous outcomes from birth certificates compared to maternal recall

Difference from maternal recall	Birth certificate	
	n	%
Birth weight		
0	0	0.0
± 50 g	2273	89.6
± 100 g	2418	95.3
± 150 g	2489	98.1
± 200 g	2538	100.0
Gestational age		
0	2655	98.9
± 1 week	2684	99.9
± 2 weeks	1	100.0

**Table 6:** Continuous outcomes from birth certificates and maternal interview compared to medical records

Difference from maternal recall	Birth certificate		Maternal recall	
	n	%	n	%
Birth weight				
0	0	0.0	0	0.0
± 50 g	1764	98.6	1572	90.9
± 100 g	1774	99.1	1650	95.4
± 150 g	1781	99.5	1698	98.2
± 200 g	1790	100.0	1729	100.0
Gestational age				
0	1906	98.9	1902	98.9
± 1 week	1925	99.9	1921	99.9
± 2 weeks	1927	100.0	1923	100.0
Apgar score (1-min)				
0	1862	97.1	–	–
± 1	1900	99.1	–	–
± 2	1906	99.5	–	–
± 3	1909	99.8	–	–
± 4	1913	100.0	–	–
Apgar score (5-min)				
0	1886	98.4	–	–
± 1	1907	99.5	–	–
± 2	1914	99.9	–	–
± 3	1915	99.9	–	–
± 4	1916	100.0	–	–

## Discussion

In this study comparing birth certificate, maternal interview data, and medical records from women who participated in IHIPS, we found excellent agreement between maternal interview and/or birth certificates compared to medical records for key labor and delivery outcomes: gestational age (in weeks), preterm birth, post-term birth, infant sex, Apgar scores, mode of delivery, birth weight (grams), and low birth weight. Birth certificate data was in moderate agreement with medical records regarding labor induction, but poor agreement with maternal interview regarding smoking and alcohol use during pregnancy. Further, all three data sources were in poor agreement as to whether or not the women gained weight during their pregnancy (kappa 0.07-0.26). Inaccurate reporting of either the exposure and/or outcome can result in misclassification of study participants, biasing study results. Understanding the most accurate, and feasible, data source for a given research question is imperative to minimization of bias.

Birth weight and gestational age are important metrics for assessing pregnancy outcomes, but they are also key characteristics in studies of how early life events from the perinatal period impact future health and disease. We found excellent agreement between medical charts and maternal interview for both birth weight and gestational age, with 95.4% of birth weights recalled within ±100 g and 98.9% exact gestational age agreement compared to medical record data. Our results are similar to those of prior validation studies of birth weight and gestational age [2–5, 7, 8], including those performed within US populations [1, 3, 4]. Two prior studies comparing maternal interview and birth registry data also found high agreement for measures of birth weight and gestational age, similar to our results [11, 12]. To our knowledge, this is the first study to validate birth certificate data with medical records for birth weight or gestational age in the United States, however the HUNT study in Norway did find a positive predictive value of 92% for preterm birth between the Medical Birth Registry of Norway and hospital records [15].

Additional characteristics surrounding labor and delivery can also be identified from multiple data sources, however, the validity varies widely depending upon the variable(s) of interest. Similar to our findings, prior validation studies have found excellent agreement between medical records and maternal recall for mode of delivery [1, 4–6, 8–10, 18], although, we did observe a lower specificity between maternal recall and birth certificate data. One prior study evaluated 1-min and 5-min APGAR score agreement, by category, between maternal recall and medical records and found a low agreement (kappa = 0.34 and 0.38, respectively) [6]. While women in IHIPS were not asked to recall their infant’s APGAR scores, we did collect this information from both the birth certificates and medical records and found very high agreement between the two data sources with > 97% perfect agreement and ≥ 99.1% agreement within one point.

To our knowledge, only two studies have assessed the validity of labor induction between birth certificates and medical records. A study using birth certificate and medical record data from PRAMS participants in New York City and Vermont found similar rates of labor induction between the data sources in both states, although the birth certificates consistently showed a 1-2% lower prevalence of labor induction [19]. A second study using data from two Hillsborough County hospitals in Florida, found similar rates of labor induction in one hospital between the birth certificates and medical records (*n* = 1168 and 1158); however, birth certificates from deliveries at a second hospital indicate nearly 20x the number of labor inductions compared to the medical records (*n* = 1207 versus 63, respectively) [14], suggesting potential wide variability in the accuracy of recording labor induction. While we observed an overall moderate agreement between birth certificates and medical records (kappa = 0.61), the sensitivity and PPV were lower, indicating labor induction was underreported on the birth certificates for the participants in IHIPS.

We observed very low agreement between maternal recall and birth certificates for smoking and alcohol use during pregnancy. Because the demographic information on birth certificates is usually completed by the mother soon after delivery, we would have expected the agreement to have been higher. However, the prevalence of smoking during pregnancy was reportedly higher on the birth certificates than the maternal interview (12.0 vs 11.3%) while alcohol use during pregnancy had a reportedly higher prevalence in the survey data than the birth certificates (5.5 vs 1.0%). One prior study that evaluated maternal recall of smoking and alcohol use 8-10 years postpartum also found low agreement with medical records for alcohol use during pregnancy (kappa = 0.08), but substantially higher agreement for smoking during pregnancy (kappa = 0.73) [10]. It is worth noting that the birth certificate is considered to be a legal document and individuals may be hesitant to disclose certain behaviors, such as alcohol consumption during pregnancy; this could explain the lower prevalence of alcohol consumption on the birth certificates when compared to maternal interview.

This study is unable to distinguish between reasons for imperfect recall and/or database inaccuracies. Factors that can affect the accuracy of maternal recall include: maternal knowledge of an event, the perceived importance of an event, how an event is defined, the way a question is asked on the survey or interview, time between delivery and completion of the interview, or simple memory lapses. While we assume that the information contained in the medical chart was made known to the mother, this assumption is probably not correct. Additionally, because birth certificates are completed by hospital staff using information from the medical charts, we would also assume a high agreement. However, data entry error, lack of reporting standards (e.g., birth weight reported in grams versus pounds and ounces), and missing/incomplete data could explain the observed disagreements. Because few validation studies have been performed between birth certificate and medical record data, it is unclear how generalizable our overall validation findings between birth certificates and medical chart data may be to other populations.

Despite the large study population and multiple sources of data for validity comparisons, this study is not without limitations. One limitation of the study is the heterogeneity between labor and delivery hospitals. While we do not have data as to which hospital a woman delivered her infant, the study population arose from a four-county catchment area that included several university-affiliated and private hospitals. While the birth certificate forms are standardized across the state of Iowa, medical records are not standardized. Each provider and health care system may have systematic differences in how events of labor and delivery are recorded in the patient’s chart, particularly negative findings or non-events [15]. Additionally, there may be provider differences in how birth weight is recorded (e.g., providing birth weight in grams or pounds and ounces). We also recognize that data from medical records is considered more reliable, but can and do suffer from recording errors and missing information [3, 20, 21]. Further, while the patients are responsible for completing a portion of the birth certificate upon delivery, some of the information (e.g., smoking and alcohol use during pregnancy) is still subject to recall bias and may be recorded incorrectly. Lastly, not all of the examined variables were available in sources of data and, therefore, could not be validated across all three datasets.

## Conclusions

Although medical record data are believed to be among the most accurate sources of health outcome information, their use can be impractical due to limited financial and human resources. Conversely, acquiring birth certificates is considerably less expensive and much faster. Surveying or interviewing participants is also typically less expensive than medical record abstraction and can yield information not available through medical records. Our study indicates strong overall agreement between all three data sources for most of the interrogated labor and delivery events, signifying it is plausible for medical records, birth certificates, and maternal interviews to be valid sources for data collection. Future research should focus on identifying factors that may be associated with poorer agreement between data sources and validation of other labor and delivery events not included in our study.

## Data Availability

The datasets generated and/or analysed during the current study are not publicly due to the wording of the informed consent document and agreements with the funding agency, but are available from the corresponding author on reasonable request.

## Abbreviations

CATI: Computer-assisted telephone interview
IHIPS: Iowa Health in Pregnancy Study
LBW: low birth weight
NPV: negative predictive value
PPV: positive predictive value
PTB: Preterm birth
SGA: Small-for-gestational age
